# Evaluation of the Shelf Life of Multigrain Puffed Foods Bound with Liquid Egg or Pullulan

**DOI:** 10.3390/foods15132324

**Published:** 2026-07-01

**Authors:** Atsuko Takahashi, Keiko Fujii

**Affiliations:** Department of Food Science, Faculty of Food Science and Nutrition, Japan Women’s University, 2-8-1 Mejirodai, Bunkyo-ku, Tokyo 112-8681, Japan; takahashiat@fc.jwu.ac.jp

**Keywords:** multigrain, puff, emergency food, rupture properties, glass transition

## Abstract

To evaluate the stability of multigrain puffed foods, samples bound with liquid egg or pullulan were stored at 25 °C under eight relative humidity (RH) conditions (6–94%). For shelf-life assessment, the samples were additionally stored at 25 °C and 57.6% RH in a constant-temperature chamber for 30 days. Their rupture properties, moisture sorption behavior, and starch retrogradation were examined. Pullulan-bound samples maintained their apparent elastic modulus throughout the one-month storage period. In contrast, liquid egg-bound samples exhibited a marked decrease in modulus by day 2, followed by a gradual recovery; by day 7, both sample types showed comparable values. The glass transition temperature (Tg) increased as moisture content decreased. At approximately 4% moisture content on the baking day, the glass-to-rubber transition occurred at 50–60 °C. X-ray diffraction analysis confirmed that no starch retrogradation occurred after one month of storage. Overall, these results demonstrate that multigrain puffed foods—particularly those bound with pullulan—retain their textural and structural stability during one month of storage. These findings provide a scientific basis for developing nutritionally balanced, allergen-conscious, and shelf-stable multigrain puffed products, supporting their potential use as meal-replacement-type emergency foods.

## 1. Introduction

Interest in emergency food supplies has grown in recent years owing to the increasing frequency of natural disasters. In response, the present study developed two types of multigrain puffed foods using multigrain cereals, which are rich in vitamins and minerals and have recently been re-evaluated for their nutritional value. Puffing is a promising method for incorporating whole grains into emergency foods because it produces low-moisture, lightweight products with excellent shelf life. However, because moisture adsorption leads to deterioration in texture—the primary cause of quality loss in puffed foods—evaluating their shelf life is essential.

In food engineering, drying and dehydration processes that reduce temperature and moisture content are widely used, as the physical and chemical properties of foods are strongly influenced by moisture content. Consequently, moisture sorption isotherms are commonly employed to evaluate water behavior in foods. Previous studies have examined the moisture sorption characteristics of biscuits [1] and the moisture sorption and textural properties of various baked products such as cookies, crackers, rice crackers, and wafers [2,3,4].

Many dried foods exist in a glassy state, and previous studies have demonstrated the importance of quality control based on the glass transition phenomenon [5,6]. Growing interest in glass transition stems from its ability to explain changes in physical properties, food deterioration, and shelf life.

Because emergency foods require long-term storage, new methods are urgently needed to appropriately control changes in their physical properties during storage. Although the concept of glass transition has been successfully applied to powders and baked products, its applicability to multigrain puffed foods with complex porous structures has not been sufficiently investigated. Furthermore, the specific moisture adsorption characteristics of puffed foods and the effects of binding agents remain unclear. Therefore, in this study, two types of multigrain puffed foods, bound and molded using liquid egg and pullulan preparations, were produced to evaluate their shelf life. Their rupture properties, moisture sorption characteristics, and glass transition temperatures (Tg) were measured under eight relative humidity (RH) conditions (6–94%) at 25 °C.

Liquid egg forms an irreversible protein network upon heating, providing high structural strength as a binder; however, it contains allergens. In contrast, pullulan is a neutral polysaccharide with excellent film-forming ability, moisture resistance, and no allergenicity, making it suitable as an allergen-friendly binder for emergency foods. A comparative evaluation of these binders enables clarification of how different binding mechanisms influence moisture sorption behavior, glass transition, and mechanical properties during storage.

Additionally, samples stored at 25 °C and 57.6% RH were evaluated for changes in physical properties based on rupture characteristics. The starch retrogradation of multigrain puffed foods was also analyzed using X-ray diffraction (XRD). This study aims to elucidate the relationship between glass transition induced by moisture adsorption during storage and the retention of crispness in puffed foods, thereby contributing to the scientific basis for designing nutritionally balanced and shelf-stable emergency foods.

## 2. Materials and Methods

### 2.1. Materials and Sample Preparation

#### 2.1.1. Materials

Three nutritionally superior grains with different particle sizes were selected for the multigrain puffed samples: germinated brown rice (domestic), adlay (Iwate Prefecture), and amaranth (Iwate Prefecture) [7]. Each grain was puffed by Sekizawa Shoten Co. (Saitama, Japan) using a grain puffing machine (Koyo Machinery Manufacturing Co., Ltd., Hiroshima, Japan). The pressure vessel was preheated to 230 °C. Each grain (2 kg) was loaded into the vessel, pressurized to 1.0 MPa, and rapidly depressurized to induce expansion. Puffing was performed under these conditions. The heating temperature was 240 ± 20 °C, and the heating time was approximately 7 min.

Two types of binders were used. The first was liquid egg (commercially available, laid within one week), a protein-based ingredient commonly used as a binder in foods such as hamburgers and valued for its nutritional properties. Because direct addition of liquid egg was expected to cause excessive hardening, it was whipped before use. The second binder was selected from an allergy-friendly perspective and consisted of pullulan (Nagase Vita Co., Ltd., Okayama, Japan) and the water-soluble dietary fiber isomaltodextrin (Fiberixa; Nagase Vita Co., Ltd., Okayama, Japan). Pullulan is a water-soluble polysaccharide produced extracellularly from starch by the black yeast *Aureobasidium pullulans*. Its sugar composition consists exclusively of glucose, similar to starch. Its safety has been confirmed through various tests, and it is widely used in the food industry as a thickener, stabilizer, and binder [8]. Aqueous pullulan solutions exhibit low viscosity, high stability against pH and heat, and strong adhesive properties [8,9]. Therefore, pullulan was selected as a suitable binding agent for multigrain puffed foods. Fiberixa is a novel water-soluble dietary fiber (isomaltodextrin) [10] with minimal color, taste, and odor, making it highly versatile in food applications. In this study, it was used not only for its allergy-friendly properties but also for its ability to impart a crispy texture.

Powdered granulated dashi (Higashimaru Shoyu Co., Ltd., Hyogo, Japan) was used as a flavoring agent.

#### 2.1.2. Preparation of Multigrain Puffed Foods

(A) Preparation of Binders

To prepare the egg-based binder, one egg was cracked into a bowl, the chalaza was removed, and 15 g of liquid egg was whipped using a hand mixer (HTM-5J; Cuisinart Sanei Co., Ltd., Tokyo, Japan) at 650 rpm for 2 min to create a foam.

To prepare the allergy-friendly binder, 15 g of a prepared solution was used. This solution was made by dissolving 10 g of pullulan and 5 g of isomaltodextrin (Fiberixa) in 100 g of distilled water. The mixture was stirred with a hand mixer at 650 rpm for 1 min and stored overnight in a refrigerator at 5 °C.

(B) Preparation of Multigrain Puffed Foods

Based on preliminary experiments, a mixture of adlay, germinated brown rice, and amaranth puffs (cf. 2.1.1) was prepared at a weight ratio of 2:2:1 (10 g:10 g:5 g). After thorough mixing, the blend was heated in a 600 W microwave oven for 1 min to remove excess moisture.

The binder (60% *w*/*w* relative to the puff mixture) and powdered granulated dashi (6% *w*/*w*) were added and rapidly mixed. The mixture was molded into pellets using a glass mold (20 mm diameter × 12 mm height).

After molding, the samples were baked in an oven (HEALSIO AX-GX2; SHARP Corporation, Osaka, Japan) preheated to 120 °C. Preliminary experiments tested heating at 110 °C for 30, 40, and 50 min; because 40 min yielded optimal results, samples were baked at this temperature for 40 min. After baking, the samples were cooled at room temperature (25 °C) for 30 min and immediately sealed in airtight containers for subsequent measurements.

### 2.2. Humidity Control and Storage Conditions

Multigrain puffed foods prepared with liquid egg and pullulan-based binders were stored in sealed desiccators maintained at eight RH levels (6–94%) at 25 °C [11]. This range covers the full spectrum of water activities relevant to dried foods, from low-humidity conditions where samples remain in a glassy state to high humidity conditions where moisture-induced plasticization becomes dominant. Samples were stored for 5–12 days until the change in sample mass was less than 0.1% over 24 h, which was defined as moisture equilibrium.

RH was controlled using saturated salt solutions (Kanto Chemical Co., Inc., Tokyo, Japan): 6.7% (LiBr), 11.3% (LiCl), 32.8% (MgCl_2_), 43.2% (K_2_CO_3_), 57.6% (NaBr), 75.3% (NaCl), 84.3% (KCl), and 93.6% (KNO_3_).

For shelf-life evaluation, samples were stored at 25 °C and 57.6% RH in a constant-temperature chamber for 30 days. After storage, rupture properties and starch retrogradation were analyzed.

### 2.3. Moisture Uptake Characteristics

#### 2.3.1. Determination of Moisture Content

Moisture content was measured using a halogen moisture analyzer (MB45; Mettler Toledo Co., Ltd., Tokyo, Japan). Approximately 1.5–3.0 g of sample was heated at 145 °C until the rate of mass change decreased to 1 mg per 120 s, which was defined as constant weight.

Moisture content on a dry basis (D.B.) was calculated as follows [11,12]:

$$\mathrm{Moisture}\;\mathrm{content}\;(\%\;D.B.)=\frac{\mathrm{Weight}\;\mathrm{at}\;\mathrm{moisture}\;\mathrm{adsorption}\;\mathrm{equilibrium}\times \mathrm{Moisture}\;\mathrm{content}\;\mathrm{at}\;\mathrm{equilibrium}}{\mathrm{Weight}\;\mathrm{at}\;\mathrm{equilibrium}\times (1-\mathrm{Moisture}\;\mathrm{content}\;\mathrm{before}\;\mathrm{adsorption})}$$

#### 2.3.2. Water Activity

Water activity (*A_w_*) was measured using a water activity analyzer (LabMASTER-aw Standard; Novasina AG, Lachen, Switzerland). Approximately 1 g of finely ground sample was placed in a sample cup and equilibrated at 25 °C before measurement. All measurements were performed in triplicate.

#### 2.3.3. Moisture Sorption Isotherms and Monolayer Moisture Content

Moisture sorption isotherms were constructed by plotting equilibrium moisture content (D.B.) against water activity at 25 °C.

The Brunauer–Emmett–Teller (BET) equation was applied within the appropriate water activity range to estimate monolayer moisture content [13]:

$$\frac{A_{w}}{M(1-A_{w})}=\frac{\left(C|1\right)}{CM_{m}}A_{w}+\frac{1}{CM_{m}}$$

where $A_{w}$ is water activity, M is moisture content (g/100 g dry matter), $M_{m}$ is monolayer moisture content (g/100 g dry matter), and C is a constant determined by food properties and temperature.

### 2.4. Rupture Properties After Moisture Adsorption

Rupture properties were measured using a rheometer (RE-3305; Yamaden Co., Ltd., Tokyo, Japan) with a cylindrical acrylic plunger (diameter 40 mm). The compression ratio was 80%, and the crosshead speed was 1 mm/s.

From the stress–strain curves, rupture stress, rupture strain, rupture energy, and apparent elastic modulus were calculated [14]. Crispness index was determined as the sum of the absolute values of negative stress differentials divided by the number of rupture events [15,16]. Chewiness was estimated by dividing total rupture energy by the number of rupture peaks detected in the force–deformation curve. All measurements were conducted at 25 °C (*n* = 10).

### 2.5. The Glass Transition Temperature Measurement by DSC

Tg was determined by differential scanning calorimetry (Diamond DSC; PerkinElmer Japan Co., Ltd., Yokohama, Japan). Samples were ground for 30 s, and 15–30 mg was sealed in a stainless-steel large-volume pan.

Measurements were conducted from −20 to 120 °C at a heating rate of 10 °C/min under a nitrogen atmosphere [17]. Tg was determined from the midpoint of the heat capacity change.

### 2.6. XRD Analysis of Starch Retrogradation

Samples were ground and washed with approximately three volumes of ethanol, followed by filtration. This procedure was repeated three times. The residue was washed twice with acetone and air-dried.

The dried powder was compressed into tablets (60 N; 3 min) using a hydraulic press (Riken Power; Riken Kiki Co., Ltd., Tokyo, Japan). Tablets were mounted in an aluminum sample holder (50 mm × 35 mm) for XRD analysis.

XRD measurements were performed using a diffractometer (MiniFlex6000; Rigaku Corporation, Akishima, Japan) under the following conditions: Cu Kα radiation (λ = 1.54 Å), 40 kV, 15 mA, scan range 2θ = 4–40°, and scan speed 20°/min.

To evaluate starch retrogradation, the relative crystallinity index was calculated as the ratio of diffraction intensity at 2θ = 17° to that at 2θ = 20°, as previously described [18,19,20].

### 2.7. Statistical Analysis

Statistical analyses were performed using Microsoft Excel. Differences between the two sample groups (liquid egg-bound and pullulan-bound samples) were evaluated using Student’s t-test, with significance set at *p* < 0.05.

For comparisons within the same sample type, differences among eight humidity conditions and six storage durations were analyzed using one-way analysis of variance (ANOVA). When significant main effects or interactions were detected, Tukey’s multiple comparison test was applied. When Tukey’s test could not be performed due to sample-specific limitations, Dunnett’s test was used instead. A significance level of *p* < 0.05 was adopted for all analyses.

## 3. Results and Discussion

### 3.1. Moisture Sorption Characteristics

**Figure 1:**
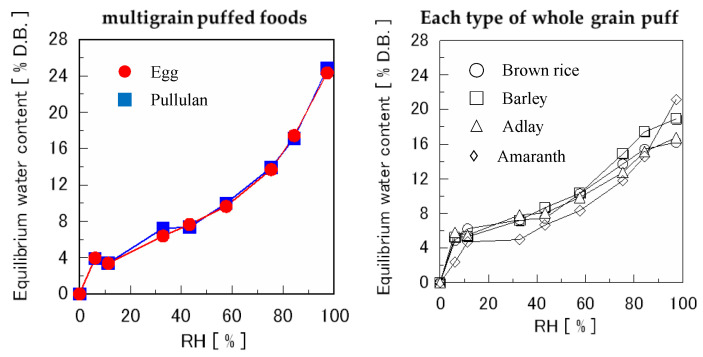
Water sorption isotherm of multigrain puffed foods and each type of whole grain puff.

Various puffed whole grains were conditioned at eight RH levels (6–94%) to determine their equilibrium moisture content. The moisture sorption isotherms of the puffed grains are shown in Figure 1. Both liquid egg- and pullulan-bound samples exhibited the characteristic inverted S-shaped curve typical of food moisture sorption isotherms. The water content up to the first inflection point represents bound water with a single-molecule adsorption capacity. According to the general understanding in the literature [21], drying to this moisture level may suppress most food deterioration reactions, including microbial spoilage. However, moisture levels below this point increase susceptibility to oxidation reactions. Therefore, drying to the moisture content around this inflection point is considered desirable.

The moisture sorption isotherms of the liquid egg- and pullulan-bound samples exhibited an inflection point at approximately 4% moisture content. This value is slightly lower than the 4–6% range previously reported for individual whole-grain puffs [11]. Thus, the present results indicate that the monolayer-equivalent moisture region for the bound puffed foods occurs around 4%. This value may serve as an approximate reference point for drying, although it should not be interpreted as a strict optimal target. The mechanism underlying the slightly lower inflection point cannot be conclusively determined from the present data. One possible explanation is that the binder coating may alter initial adsorption behavior, enabling monolayer adsorption to occur at a lower moisture content. However, this remains a hypothesis consistent with the observed trend rather than a conclusion directly demonstrated by the dataset.

RH values in the range of 5–35% were fitted to the BET equation [22]. Liquid egg-bound samples exhibited a monolayer adsorption capacity of 13.2 g/100 g dry solid, whereas pullulan-bound samples exhibited a capacity of 16.5 g/100 g dry solid, indicating a higher monolayer adsorption capacity for the pullulan preparation. The monomolecular adsorption capacities of agar sticks and dried kanpyo are 11 and 14 g/100 g dry solid, respectively [23], and similar values were obtained in this study. In a previous study [24], the monomolecular adsorption capacity of commercially available confectionery products, such as cookies, crackers, and rice crackers at 7.6–22% RH (calculated using the Guggenheim–Anderson–de Boer method), was 3.9–7.1 g/100 g dry solid. Confectionery with a high degree of gelatinization exhibits a high monomolecular adsorption capacity and improved mechanical properties. However, the multigrain puffed foods in this study showed values approximately 2–4 times higher than those of commercial confectionery. This difference was possibly because, unlike confectionery made from wheat or rice flour, multigrain puffs retain their grain shape during puffing, with the outer husk intact.

In a previous sensory evaluation [25], pullulan-bound samples showed significantly greater chewiness than liquid egg-bound samples, with some respondents noting a greater sense of moisture in the pullulan-bound samples. This was speculated to be influenced by the higher monolayer adsorption capacity of pullulan-bound samples. A previous study [26] reported that consumers perceive texture changes when moisture content exceeds the monolayer adsorption amount. However, the monolayer adsorption amount determined using the BET method also correlates with the crispness of dried foods. In this study, the higher monolayer adsorption capacity of pullulan-bound samples may have contributed to the perceived chewiness.

### 3.2. Rupture Properties of Various Multigrain Puffed Foods After Moisture Adsorption

#### 3.2.1. Stress–Strain Curves and Derivatives of Humidified Multigrain Puffed Foods

**Figure 2:**
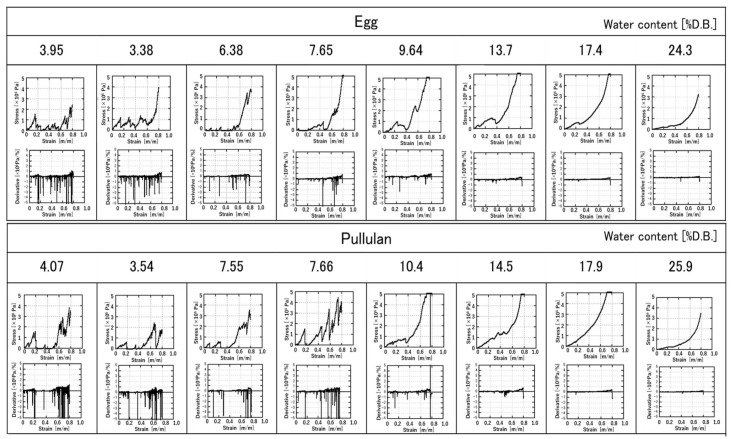
Effect of equilibrium water content on stress–strain curves and derivative curves of each multigrain puffed food (25°C).

Figure 2 presents the stress–strain curves and their derivatives for multigrain puffed foods bound with liquid egg and pullulan and humidified under eight RH levels at 25 °C. In the differential curve, the degree of stress decay after rupture indicates rupture impact, represented by the negative stress derivative value. A larger negative value suggests a greater rupture impact. Additionally, the number of negative stress derivative values reflects the number of ruptures, allowing analysis of the crispy texture of puffed foods using both stress–strain and differential curves.

For all whole-grain puffed foods, multiple distinct rupture points were observed in the stress–strain curve at moisture content < 8%, indicating brittle rupture behavior. The negative stress derivative values at these points were large and numerous, characteristic of dry foods during rupture. Multiple cracks formed under impact, leading to disintegration.

Samples with a moisture content of approximately 10% exhibited few rupture points and small negative stress derivatives, indicating reduced crisp fracture behavior. At moisture contents ≥ 13%, no distinct rupture points were observed, indicating a transition from brittle to ductile rupture. Large negative stress derivatives were absent, suggesting minimal crack formation. A previous study [27] reported similar findings: muffins with moist textures exhibited smooth load–strain curves without distinct breakpoints, whereas crisp cookies exhibited multiple breakpoints and large differential changes. These observations are consistent with the present results.

#### 3.2.2. Rupture Characteristics

**Figure 3:**
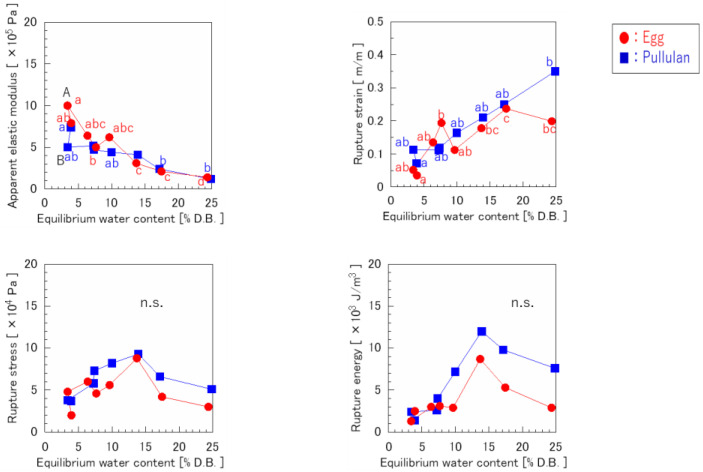
Effect of equilibrium water content on the rupture properties of each multigrain puffed food. Different capital letters denote significant differences (*p* < 0.05) between egg and pullulan. Different lowercase letters denote significant differences (*p* < 0.05) within the same sample. “n.s.” indicates no significant difference.

Figure 3 shows the rupture characteristics of multigrain puffed foods bound and molded using liquid egg and pullulan preparations and humidified under eight RH levels at 25 °C. The apparent elastic modulus decreased as moisture content increased for both sample types. A previous study reported that the apparent elastic modulus of commercial cookies, biscuits, crackers, and rice crackers decreased with increasing environmental humidity, with a rapid decline around 43% RH [28]. A similar trend was observed here. However, although liquid egg-bound samples showed a significant decrease in apparent elastic modulus in the 3–14% moisture range, pullulan-bound samples exhibited little change within this range.

Rupture strain increased with increasing moisture content in both sample types, indicating more flexible structures. In the dry state, compression caused immediate cracking, resulting in low rupture strain. As moisture content increased, the samples became more resistant to rupture, leading to higher rupture strain values.

Rupture stress increased with increasing moisture content in both sample types, peaking at approximately 14% moisture content, after which it decreased. Rupture energy also peaked at approximately 14% moisture content, showing a trend similar to rupture stress. For commercial confectionery, changes in hardness due to moisture adsorption are minimal under low-humidity conditions, where products remain dry and exhibit brittle rupture [28]. Similar results were obtained in this study. However, due to large variation in rupture stress and rupture energy, no statistically significant differences were detected.

The mechanical behavior shown in Figure 3 reflects moisture-dependent changes in the structural integrity of the puffed matrix. At low-moisture contents, the cell walls remain rigid and brittle, resulting in low rupture strain and low energy adsorption. As moisture increases, water penetrates the porous network and softens the cell walls, allowing the structure to deform more before failure. This explains the monotonic increase in rupture strain. The non-monotonic behavior of rupture stress and rupture energy, both peaking around 14% moisture content, suggests an intermediate regime where the structure becomes temporarily tougher. In this region, the cell walls retain sufficient stiffness to support load, while added moisture enhances the ability of the porous network to redistribute stress and blunt crack propagation. Once moisture exceeds this optimal range, the structure becomes overly softened, reducing load-bearing capacity and fracture stress. These results indicate that the rupture properties of puffed foods are governed by the balance between structural rigidity and moisture-induced softening within the porous matrix.

**Figure 4:**
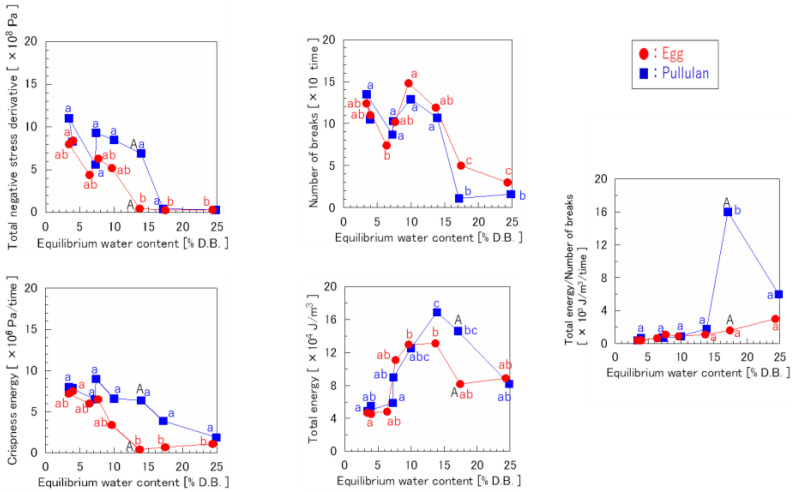
Effect of equilibrium water content on the crispy characteristics of each multigrain puffed food. Different capital letters denote significant differences (*p* < 0.05) between egg and pullulan. Different lowercase letters denote significant differences (*p* < 0.05) within the same sample.

Figure 4 shows the crispness measurements of various multigrain puffed foods. As the negative stress derivative value indicates the degree of stress decay after rupture, the total negative stress derivative values were calculated. Pullulan-bound samples exhibited higher values up to approximately 15% moisture content compared with liquid egg-bound samples. At moisture contents > 17%, the values approached zero for both sample types, indicating a transition from brittle rupture to a more pliable texture resembling wet rice crackers.

The number of ruptures showed a trend similar to that of the total negative stress derivative values. However, at moisture content ≥ 10%, liquid egg-bound samples exhibited a higher number of ruptures than pullulan-bound samples, suggesting that liquid egg-bound samples retain crispness better at higher moisture levels.

Crispness was also quantified using a crispness index, defined as the sum of negative stress derivative values divided by the number of fracture events. A previous study [16] on rusks prepared with millet flour reported that samples with lower crispness index values were evaluated as having higher crispness (“saku-saku” texture) in sensory tests. However, in humidity-conditioned samples such as those in the present study, increasing moisture content caused a drastic reduction in both total negative stress derivative values and the number of breaks. Consequently, in the high-moisture region, the crispness index markedly decreased. Additionally, pullulan-bound samples exhibited higher crispness index values than liquid egg-bound samples.

Pullulan is a polysaccharide that readily forms a glassy matrix and may maintain structural integrity without rapid collapse even when some moisture is absorbed. As a result, fine pore structure and brittleness are likely preserved, maintaining a high crispness index. In contrast, egg proteins readily interact with water and tend to swell and become plasticized upon moisture adsorption. Therefore, even at relatively low-moisture contents, the structure may transition toward a more flexible and less brittle state, resulting in lower crispness index values.

Although no sensory evaluation was conducted in the present study and definitive conclusions cannot be drawn, the results do not fully agree with those of the previous study [16]. Rather, the crispness index appears to represent a “crunchy” (“zaku-zaku”) texture rather than a light crisp (“saku-saku”) texture when higher values are obtained, and the loss of crunchiness appears to occur as moisture content increases. Based on this assumption, further investigations combining instrumental measurements with sensory evaluation are planned.

The chewiness of multigrain puffed foods was also evaluated by dividing the total energy from the stress–strain curve by the number of breaks. Pullulan-bound samples at 17% moisture content showed a significantly higher value, possibly because the total energy was high (approximately 14 × 10^4^ J/m^3^) while the number of breaks was the lowest (11). For other samples, chewiness tended to increase with moisture content. As samples absorbed moisture, they became damp, making crisp cracks less likely to form, reducing the number of breaks, and generating a chewy texture. Although no difference was observed between the two sample types at moisture content < 10%, pullulan-bound samples exhibited higher values at higher moisture contents, suggesting greater chewiness.

However, using total energy divided by the number of breaks as a proxy for “chewiness” is an indirect and non-standard method. Ideally, this instrumental evaluation should be validated through comparison with sensory panel data. Therefore, future work will include combined instrumental and sensory evaluation.

### 3.3. Tg

**Figure 5:**
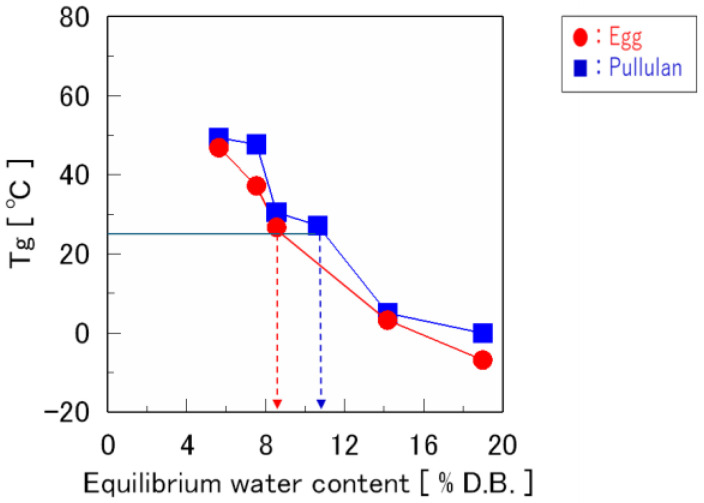
Glass transition temperature of each multigrain puffed food.

Figure 5 shows the Tg values of various multigrain puffed foods conditioned at 25 °C. The Tg ranged from −7 to 53 °C and decreased as equilibrium moisture content increased.

Regarding the rupture characteristics of humidity-conditioned multigrain puffed foods, the crisp texture region corresponds to a state in which polymer chains lose fluidity while retaining an irregular structure, resulting in a glassy state [28]. The decrease in apparent elastic modulus with increasing moisture content is associated with food softening and transition to a rubbery state. Both moisture content and temperature influence the glassy to rubbery transition [29]. An increase in either factor enhances molecular mobility, reducing resistance to deformation. This decrease in viscosity and Tg causes the material to soften and become plastic—a process known as plasticization [30].

In this study, both multigrain puffed foods exhibited maximum rupture stress and rupture energy at approximately 14% moisture content, followed by a decrease as moisture content increased. This behavior reflects moisture-induced softening, which shifts the material away from the glass transition region and into the rubbery state.

Previous reports have indicated that the glass transition point for dough and baked goods occurs at a moisture content of 14–22% at 15–25 °C [31]. Commercial toast bread transitions from a glassy to a rubbery state at 11.1% moisture content at 25 °C, with crispness completely lost at 9.3% moisture content [32]. In this study, the boundary between the glassy and rubbery states at 25 °C was approximately 9% D.B. for liquid egg-bound samples and approximately 11% D.B. for pullulan-bound samples. Although rupture stress and rupture energy reached maximum values at approximately 14% D.B., the Tg values in Figure 5 indicate that this moisture level corresponds to the rubbery state at 25 °C. Therefore, the observed maxima do not represent the glass–rubber transition point itself but instead reflect structural relaxation occurring immediately after the transition induced by moisture adsorption. In contrast, the number of breaks peaked at approximately 10% D.B., which corresponds closely to the boundary region between the glassy and rubbery states at 25 °C.

In wafer research [4], water plasticization occurs beyond the monolayer adsorption capacity. From the perspective of the 6% D.B. monolayer moisture content calculated using the BET equation, the decrease in elastic modulus indicated that water acted as a plasticizer. Wafers remained in a glassy state at room temperature (20 °C) up to a 11% moisture content, and a moisture content of 6–11% D.B. was considered optimal for achieving a moderately crisp texture without excessive brittleness. In this study, a decrease in apparent elastic modulus was observed at 4% D.B. for all multigrain puffed foods (Figure 3). Furthermore, the onset of the moisture sorption isotherm corresponded to 4% D.B., suggesting that drying to this moisture level is desirable. The Tg at 25 °C was 9% D.B. for liquid egg-bound samples and 11% D.B. for pullulan-bound samples. Therefore, moisture contents of 4–9% D.B. (liquid egg) and 4–11% D.B. (pullulan) were found to maintain crisp texture in the puffed foods.

A moisture content of approximately 4% is generally targeted for finished puffed foods to maintain crispness [33]. In this study, the Tg at 4% D.B. was approximately 55–60 °C, confirming that a sufficiently crisp glassy state can be maintained even during summer storage.

### 3.4. Effects of Storage on Moisture Content and Water Activity of Various Multigrain Puffed Foods

**Figure 6:**
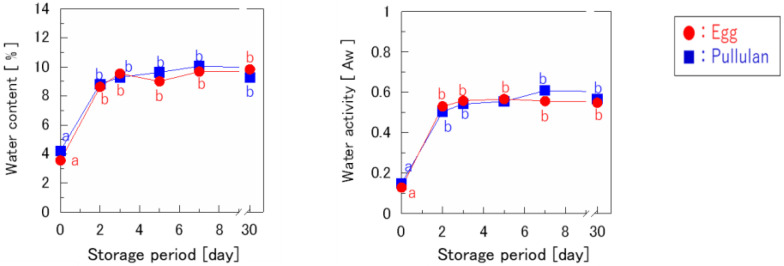
Effect of storage period on the water content and water activity of each multigrain puffed food. Different lowercase letters denote significant differences (*p* < 0.05) within the same sample.

Figure 6 shows the moisture content and water activity of multigrain puffed foods stored for 30 days at 57.6% RH. Moisture content for both sample types was approximately 4% immediately after baking. By day 2, moisture content increased to approximately 9%, more than double the initial value, but showed little change thereafter, remaining below 10% after one month. No differences were observed between binder types. Water activity followed a similar pattern. Immediately after baking, both sample types exhibited a water activity of approximately 0.15, which increased to 0.5 by day 2. After one month, water activity remained between 0.5 and 0.6.

A previous study [34] reported that rice puffs produced by baking or extrusion exhibit a water activity of 0.5 and an equilibrium moisture content of 8–10%. Combined with sensory evaluation results [35,36,37], the critical moisture content for crispness corresponds to a water activity of 0.45–0.55. In this study, the water activity of stored multigrain puffed foods was close to these reported values. Storage conditions simulated typical indoor environments (50–60% RH at 25 °C). After one month, water activity slightly exceeded the upper limit of the critical crispness range. Therefore, adding desiccants and using airtight containers or zipper bags may help maintain the desirable 4% moisture content after baking.

### 3.5. Effects of Storage on the Breaking Properties of Various Multigrain Puffed Foods

**Figure 7:**
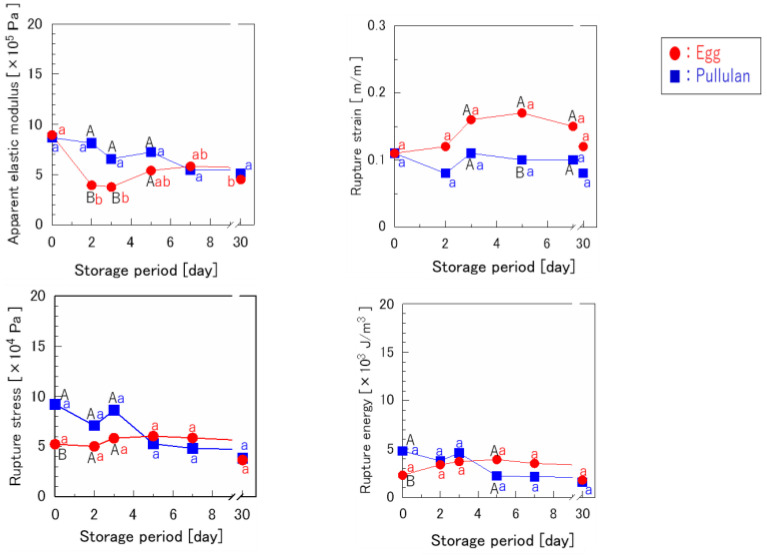
Effect of storage period on rupture properties of each multigrain puffed food. Different capital letters denote significant differences (*p* < 0.05) between egg and pullulan. Different lowercase letters denote significant differences (*p* < 0.05) within the same sample.

Figure 7 shows the effects of storage on the breaking properties of multigrain puffed foods. The apparent elastic modulus showed no significant changes during one month of storage for pullulan-bound samples. In contrast, liquid egg-bound samples exhibited a significant decrease from immediately after baking to day 2, followed by a gradual increase, reaching values similar to those of pullulan-bound samples by day 7.

Rupture strain of pullulan-bound samples was slightly lower than that of liquid egg-bound samples, but both types showed little change over time, fracturing at approximately 10% deformation.

Immediately after baking, rupture stress and rupture energy were higher for pullulan-bound samples. However, from day 2 onward, no significant differences were observed between formulations. Storage for 30 days had little effect on rupture properties.

**Figure 8:**
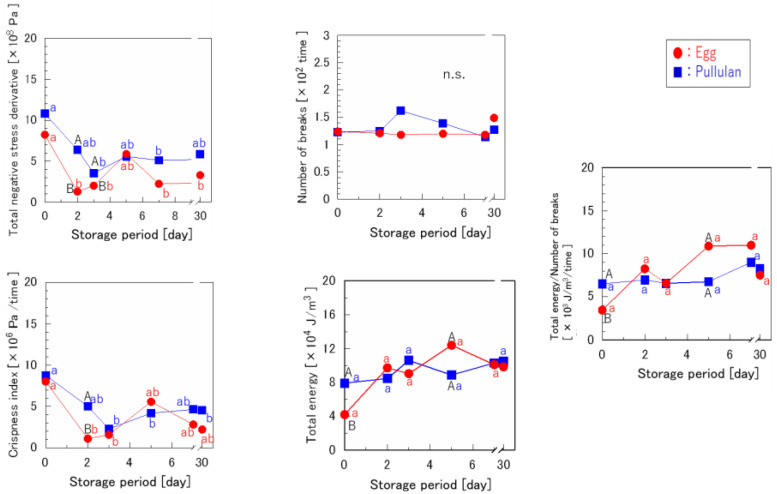
Effect of storage on crisp characteristics of each multigrain puffed food. Different capital letters denote significant differences (*p* < 0.05) between egg and pullulan. Different lowercase letters denote significant differences (*p* < 0.05) within the same sample. “n.s.” indicates no significant difference.

Figure 8 shows the effects of storage on rupture characteristics and crisp texture. The total negative stress derivative value was high immediately after baking but decreased significantly by day 2, likely due to moisture adsorption and loss of brittleness. Pullulan-bound samples generally exhibited higher values than liquid egg-bound samples.

The crispness index showed similar trends, as the number of breaks remained nearly constant over time. Pullulan-bound samples exhibited higher crispness index values than liquid egg-bound samples, except on day 5.

The value obtained by dividing total rupture energy by the number of breaks was higher for pullulan-bound samples immediately after baking, suggesting greater chewiness [25]. However, over time, liquid egg-bound samples exhibited higher values, indicating increased chewiness during storage. After one month, both sample types showed nearly identical values.

### 3.6. XRD Analysis of Starch Retrogradation of Various Multigrain Puffed Foods

**Figure 9:**
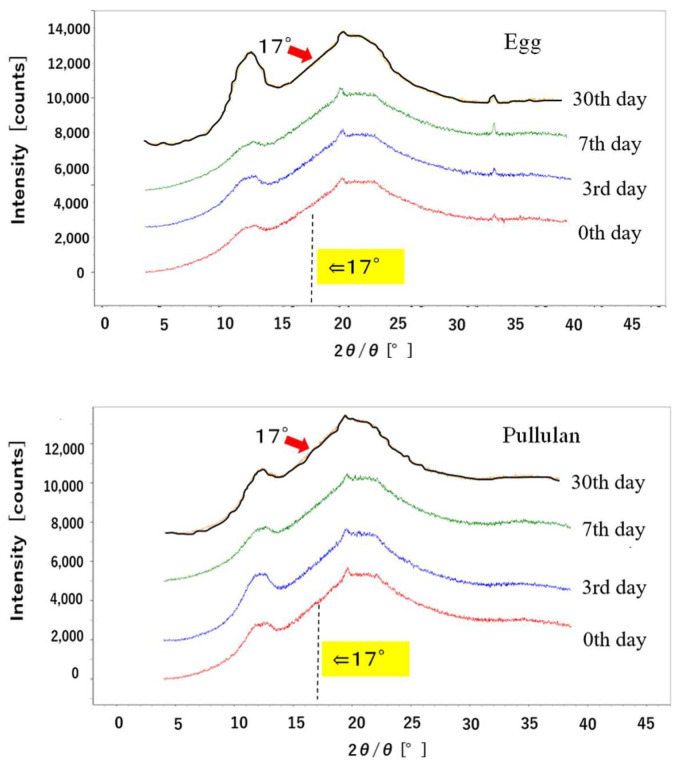
X-ray diffraction patterns during storage of each multigrain puffed food.

**Figure 10:**
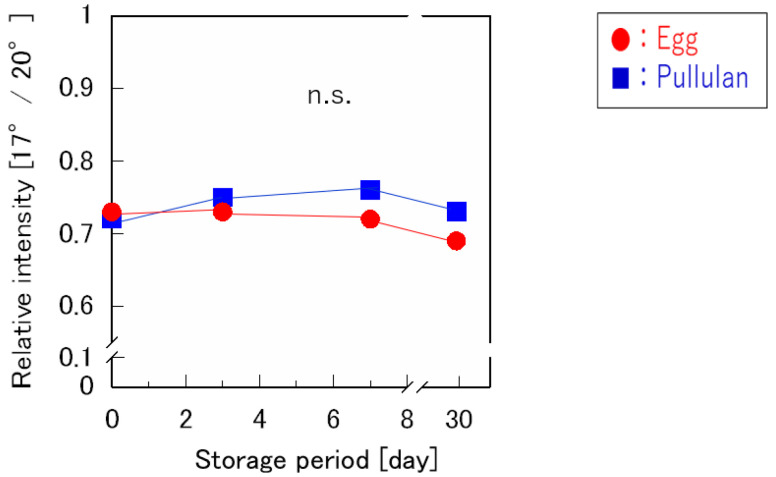
Relative X-ray intensity during storage of each multigrain puffed food. “n.s.” indicates no significant difference.

XRD patterns of samples stored for 3, 7, and 30 days are shown in Figure 9. The relative intensities at 2θ = 17° are shown in Figure 10.

Both liquid egg and pullulan-bound samples showed a peak near 2θ = 12–13° at all storage periods. This peak became significantly larger in liquid egg-bound samples after one month. This peak corresponds to the diffraction peak at 13.1° [18,19], indicating the formation of amylose–lipid complexes. This suggests interactions between lipids in the liquid egg and amylose. Additionally, only liquid egg-bound samples showed a sharp peak near 2θ = 32°, corresponding to sodium chloride (NaCl) crystals. The exclusive appearance of this peak suggests that charged egg white proteins (e.g., ovalbumin) may have promoted salt precipitation or orientation.

Starch retrogradation is typically indicated by a peak at 2θ = 17° [20]. However, no such peak was observed in any sample stored for one month, suggesting that no detectable starch retrogradation occurred under the storage conditions used.

The differences observed between egg-bound and pullulan-bound samples cannot be attributed to grain composition, particle size, or storage conditions, as these were constant across formulations. Instead, the contrasting behaviors arise from the intrinsic properties of the binders. Liquid egg forms a protein–starch matrix whose mechanical response is influenced by moisture-induced plasticization and subsequent protein aggregation and starch retrogradation. Pullulan forms an amorphous polysaccharide matrix that undergoes continuous plasticization without network-strengthening processes. These polymer specific mechanisms explain the different storage responses.

Whipping eggs may introduce variability in freshness and air incorporation. Additionally, eggs were used after whipping, whereas pullulan was used after stirring, resulting in differences in solid and moisture content. However, this study focused on the functional role of whipped egg proteins in binding multigrain puffed foods.

Although some parameters did not show statistically significant differences, overall trends indicate that mechanical properties changed differently depending on binder type. Egg-bound samples tended to show an initial decrease in apparent elastic modulus and rupture stress, followed by partial recovery, suggesting moisture-induced softening and structural rearrangement. Pullulan-bound samples generally exhibited a gradual decline, consistent with continuous plasticization of the amorphous polysaccharide matrix. These tendencies, though not always statistically significant, support the interpretation that the two binder systems respond differently to moisture during storage.

Based on the 30-day storage results, binder choice has clear implications for emergency food applications. Pullulan-bound samples exhibited stable rupture properties across a wide humidity range, indicating suitability for products requiring long term texture stability. Liquid egg binding offers advantages in flavor, nutrition, and browning but is more sensitive to moisture-induced softening; thus, it is better suited for applications where low moisture can be strictly controlled or where sensory attributes other than crispness are prioritized. These findings suggest that pullulan is the more robust binder for emergency ration development, particularly when combined with moisture control strategies such as low permeability packaging and desiccants.

## 4. Conclusions

To investigate the shelf life of multigrain puffed foods, samples bound and molded using liquid egg and pullulan preparations were conditioned at 25 °C under eight RH levels (6–94%). Their rupture properties and moisture sorption characteristics were examined, and starch retrogradation was assessed by XRD analysis.

Regarding rupture properties, pullulan-bound samples maintained their apparent elastic modulus throughout one month of storage. In contrast, liquid egg-bound samples showed a significant decrease from immediately after baking to day 2, followed by a gradual increase; by day 7, the modulus reached values nearly equivalent to those of pullulan-bound samples.

Tg, which reflects the physical state of the food, increased as moisture content decreased. For samples with approximately 4% moisture content on the baking day, the glass-to-rubber transition was inferred to occur at approximately 50–60 °C.

XRD analysis revealed no peak near 2θ = 17°, an indicator of starch retrogradation, in any sample after approximately one month. These findings suggest that no detectable starch retrogradation occurred under the storage conditions used in this study.

The significance of this study lies in the systematic application of food engineering methods to the relatively unexplored field of multigrain puffed foods, thereby establishing a scientific foundation for a new category of emergency foods that simultaneously meet requirements for nutritional value, allergen safety, and shelf life. Furthermore, the development of “meal-replacement–type emergency foods” that leverage the nutritional value of multigrain and the umami of dashi stock may create new market value distinct from conventional sweet-flavored bar-type products. Because dried foods generally require a shelf life of at least six months, future work will include three- and six-month storage tests of the multigrain puffed foods. Optimal packaging conditions and storage environments will also be investigated to support practical implementation.

## Data Availability

The data presented in this study are available on request from the corresponding author.

## Abbreviations

The following abbreviations are used in this manuscript:

RH	relative humidity
Tg	glass transition temperature
XRD	X-ray diffraction
